# Korean Red Ginseng Polysaccharides Enhance Intestinal IgA Production and Barrier Function via Peyer’s Patch Activation in Mice

**DOI:** 10.3390/nu16223816

**Published:** 2024-11-07

**Authors:** Sung Jin Kim, Hae-Kyung Lee, Ki Sung Kang, Mi-Gi Lee, Myoung-Sook Shin

**Affiliations:** 1 College of Korean Medicine, Gachon University, Seongnam 13120, Republic of Korea; sungjinkim001@gmail.com (S.J.K.); kkang@gachon.ac.kr (K.S.K.); 2 Avison Biomedical Research Center, Yonsei University, Seoul 03722, Republic of Korea; 2hklee@yonsei.ac.kr; 3 Bio-Center, Gyeonggi-do Business and Science Accelerator, Suwon 16229, Republic of Korea

**Keywords:** Korean red ginseng, intestinal immune modulating, IgA, Peyer’s patches

## Abstract

**Background**: Natural products are gaining attention for their potential benefits in gastrointestinal health. Plant-derived polysaccharides are essential for boosting intestinal immunity and maintaining gut homeostasis. This study investigated the effects of Korean red ginseng polysaccharides (KRG-P) on intestinal homeostasis including IgA and SCFA production and mucosal barrier integrity. **Methods**: Mice were orally administered KRG-P at doses of 50 mg/kg or 200 mg/kg for 10 days. Fecal IgA levels were measured on days 3, 5, and 11 and IgA from cultured Peyer’s patch cells from KRG-P-treated mice were analyzed. Additionally, mRNA and protein expression levels of α-defensin, lysozyme, and E-cadherin in the small intestine were examined. Short-chain fatty acids (SCFAs) content in the cecum was also assessed. **Results**: KRG-P-treated groups showed a significant increase in fecal IgA levels on days 5 and 11, with no notable change on day 3. Cultured Peyer’s patch cells from mice demonstrated heightened IgA production. Additionally, KRG-P administration upregulated α-defensin and lysozyme mRNA expression, along with elevated protein expression of E-cadherin, α-defensin, and lysozyme, in the small intestine. KRG-P treatment also led to increased cecal SCFA levels, including acetate, butyrate, and propionate. **Conclusions**: KRG-P may promote intestinal homeostasis and host defense mechanisms by activating immune cells in Peyer’s patches, stimulating IgA production, enhancing antimicrobial peptide expression, and modulating gut microbiota metabolism through increased SCFA production.

## 1. Introduction

Panax ginseng C.A. Meyer, a perennial herb belonging to the family Araliaceae and genus Panax, is commonly used in traditional medicine, primarily for its medicinal root, termed Ginseng radix [1]. The etymology of its scientific designation is derived from the Greek words “Pan” (all) and “axos” (medicine), reflecting its historical use as a panacea [2]. For over two millennia, this herb has been a staple in traditional medicinal practices across China, Japan, and Korea and has been valued for its tonic effects [3]. Recently, it has gained international prominence as a functional food and as an ingredient in alternative and complementary medicine [4]. Red ginseng, which is processed differently from white ginseng via steaming and drying, contains distinctive biochemical components, including various biologically active ginsenosides, such as Rb1, Rb2, Rc, Rd, 20(S)-Rg3, 20(R)-Rg2, and 20(R)-Rh1 [5,6,7,8]. The pharmacological activities of ginsenosides includes the following: cognitive enhancement; improvement of early Alzheimer’s disease symptoms; amelioration of insulin resistance and diabetes; reduction in muscle atrophy and bone loss; protection against UVB-irradiated skin damage; and enhancement of NK cell activity in immunosuppressed models [5,7,9,10,11]. However, the diverse physiological functions of red ginseng are not solely attributable to its ginsenosides [12,13,14]. Extensive studies have demonstrated that ginseng polysaccharides exhibit a range of physiological activities, including anti-fatigue, immune system modulation, antiviral (specifically against influenza), antibacterial, antitumor, and antioxidant effects, as well as the capacity to regulate blood lipid and glucose levels [15,16,17,18,19,20]. Plant-derived polysaccharides play crucial roles in intestinal immune response and homeostasis. Several studies indicate they promote the growth of beneficial intestinal bacteria; increase short-chain fatty acid production, including acetic acid, propionic acid and butyric acid; enhance intestinal epithelial defense; and activate immune cells [21,22,23]. Gut-associated lymphoid tissues include Peyer’s patches, which are lymphoid tissues in the small intestine, crucial for intestinal immune responses. They capture and process antigens from the gut lumen via M cells, presenting them to immune cells. This process activates antigen-specific IgA-producing B cells, a key mechanism in mucosal immunity. Peyer’s patches also regulate T cell responses and induce oral tolerance [23,24,25]. These functions are essential for maintaining intestinal homeostasis and defending against pathogens. It has already been reported that polysaccharides stimulate gut-associated lymphoid tissues, boosting IgA production and anti-inflammatory cytokine secretion [26,27]. Also, we previously indicated that polysaccharides from red ginseng can modulate intestinal immune responses via Peyer’s patches and mitigate antibiotic-associated diarrhea by regulating the intestinal flora [27]. These findings suggest a broader spectrum of potential therapeutic applications of plant-derived polysaccharides [28]. Therefore, in this study, we analyzed the effects of KRG-P on intestinal homeostasis by IgA production using feces and Peyer’s patches immune cells, intestinal barrier proteins and the mRNA in intestinal tissues, and SCFAs contents of cecum in KRG-P administered mice.

## 2. Materials and Methods

### 2.1. Preparation of KRG-P

Korean red ginseng (KRG) extract, recognized by the Ministry of Food and Drug Safety (formerly known as the Korea Food and Drug Administration) as a healthy functional food, was provided by the Korea Ginseng Corporation (Daejeon, Republic of Korea). KRG-P was extracted and purified as previously described [27]. The extract was prepared by diluting the KRG concentrate (15 Brix) with distilled water. Precipitation was induced by adding ethanol at approximately four times the volume of the concentrate. The resulting precipitate was then separated by centrifugation at 600× *g* for 20 min at 4 °C using a Super R30 centrifuge (Hanil Scientific, Daejeon, Republic of Korea). Subsequently, the precipitates were resuspended in distilled water. Low-molecular-weight substances were removed from the precipitate through dialysis. This process was carried out using Slide-A-LyzerTM Dialysis Cassettes, 20K (ThermoFisher Scientific, Waltham, MA, USA) with a molecular weight cutoff of 20 kDa. Following dialysis, the polysaccharide fraction, designated as KRG-P, was isolated and collected after lyophilization (FDU-2100, EYELA, Tokyo, Japan).

### 2.2. Reagents

For the cell culture experiments, RPMI 1640 medium manufactured by Gibco (Grand Island, NY, USA) was selected and supplemented with 1% antibiotic solution (penicillin/streptomycin). To enrich the culture medium, Fetal Bovine Serum (FBS) ATCC 30-2020, supplied by ATCC (Manassas, VA, USA), was incorporated. Quantitative analysis of mouse IgA was conducted utilizing an ELISA kit (Catalog #88-50450-88) procured from Invitrogen (Waltham, MA, USA). Antibodies for α-defensin and lysozyme were sourced from Abcam (Cambridge, UK), while β-actin (I-19) was acquired from Santa Cruz Biotechnologies (Santa Cruz, CA, USA). For the isolation of Peyer’s patches from intestinal tissues, a Corning^®^ 70 μm Cell Strainer (Corning, NY, USA) was employed. The dialysis procedure was carried out using Slide-A-Lyzer 20K MWCO dialysis Cassettes, which were purchased from Thermo Scientific (Waltham, MA, USA).

### 2.3. Animal Experiment Design

**Figure 1:**
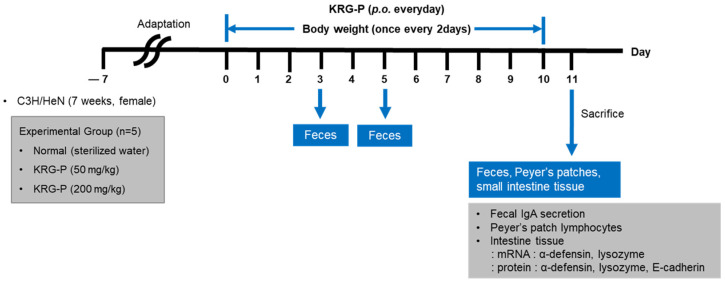
Experimental Desing for Evaluating the Intestinal Immune-Modulatory Effects of KRG-P.

The experimental protocol involving animals adhered to the guidelines set forth by the Institutional Animal Care and Use Committee (IACUC) at Gachon University (Protocol ID: GU1-2020-IA0057-00, endorsed on 6 January 2022). Seven-week-old female C3H/HeN mice, obtained from Orientbio (Seongnam, Republic of Korea), underwent a week-long adaptation period in the laboratory setting before commencing the study. The mice were housed in a regulated environment (22 ± 2 °C, 50–55% humidity) with alternating 12 h light–dark cycles and unrestricted access to sustenance and hydration. The test substance, KRG-P, was dissolved in sterile aqueous solution and administered via oral gavage daily. Two dosage groups were established, 50 mg/kg and 200 mg/kg, with the treatment regimen lasting 10 consecutive days (5 mice per group). Following the final dose administration, on day 11, fecal specimens were harvested from individual mice and promptly cryopreserved at −80 °C for subsequent IgA quantification. The animals were then euthanized using approved methods, after which Peyer’s patches were carefully excised from the small intestine under aseptic conditions (Figure 1). Concurrently, samples of small intestinal tissue were procured and stored at −80 °C for future analytical procedures.

### 2.4. Determination of IgA Content in Mouse Feces

Mouse feces were collected on the 3rd, 5th, and 11th day of oral administration and stored at −80 °C for subsequent analysis. Fecal IgA content was measured using an ELISA kit (Product #88-50450-88, Invitrogen, Waltham, MA, USA) by suspending feces in phosphate-buffered saline (PBS) at a concentration of approximately 50 mg/mL, followed by 10-fold dilution, and centrifugation to collect the supernatant.

### 2.5. Determination of IgA Content in Peyer’s Patches

After a 10-day course of KRG-P oral treatment, the experimental animals were euthanized on the subsequent day. Under aseptic conditions, Peyer’s patches were carefully extracted from the small intestinal tissue. These lymphoid aggregates were transferred to a sterile dish containing RPMI 1640 culture medium. To isolate immune cells, the patches were gently disrupted using a sterilized 100 mesh metallic sieve (Merk, Darmstadt, Germany). The resulting cellular suspension was further refined by passage through a Corning cell strainer. The purified cell population was then seeded into 96-well culture plates at a concentration of 1 × 10^6^ cells per well. The culture environment consisted of RPMI 1640 medium enriched with 10% FBS. Incubation proceeded for a duration of 5 days in a controlled atmosphere of 5% CO_2_ at 37 °C. Upon completion of the culture period, the supernatant was harvested for quantification of secretory IgA. This analysis was performed utilizing a mouse IgA ELISA kit (Product #88-50450-88) procured from Invitrogen (Waltham, MA, USA).

### 2.6. Immuno-Blotting

From mouse small intestines, protein extraction was performed using a radioimmunoprecipitation assay (RAPA) buffer. This RIPA buffer was enriched with a phosphatase inhibitor cocktail (Sigma-Aldrich, St. Louis, MO, USA), 1 mM dithiothreitol (Wako, Tokyo, Japan), and complete™ Mini Protease Inhibitor Cocktail (Roche Diagnostics Corp., Basel, Switzerland). To separate proteins from intestinal debris, the mixture underwent centrifugation (13,000 RPM, 20 min, 4 °C). Post-separation via SDS-PAGE, the proteins were electroblotted onto a PVDF membrane. This membrane was then subjected to overnight blocking with 5% skim milk solution. Subsequently, the membrane was probed with specific primary antibodies diluted in Tween-20-supplemented TBS. After three rinses with TBS-T buffer, the membrane was incubated for 2 h at an ambient temperature with horseradish peroxidase (HRP)-linked secondary antibodies. For protein visualization, Super Signal West Femto Substrate (Thermo Fisher, Waltham, MA, USA) was employed. The resulting chemiluminescence was captured using the Fusion Solo System (Vilber Lourmat, Paris, France).

### 2.7. RT-qPCR

Following euthanasia, small intestinal specimens were harvested from the mice and cryopreserved at −80 °C. For RNA extraction, these tissues underwent homogenization in RLT buffer utilizing a Biomasher apparatus (Takara, Siga, Japan). The resulting homogenate was subjected to centrifugation (8000 rpm, 20 min) in a Micro 17TR device (Hanil, Incheon, Republic of Korea). The clarified supernatant was carefully aspirated and transferred to a fresh tube. RNA isolation was accomplished using the RNeasy Mini Kit (Qiagen, Hilden, Germany), followed by quantitative assessment. Subsequently, cDNA was generated employing the RevertAid First Strand cDNA Synthesis Kit (Thermo Scientific™). Gene expression analysis was conducted via real-time quantitative PCR (RT-qPCR) using gene-specific primer pairs as detailed in Table 1. The PCR amplification process utilized either Power SYBR Green PCR Master Mix or Taqman Master Mix (Applied Biosystems, Foster City, CA, USA). Quantification of relative gene expression was performed using the ΔΔCT method on a QuantStudio-3 real-time PCR platform (Applied Biosystems).

### 2.8. Determination of SCFAs Content

Following the 10-day oral administration period, the cecal contents of the mice treated with KRG-P were processed using methanol to assess SCFAs production. SCFAs analysis was conducted using the Zhao et al. method [29]. Here, 200 mg of cecum content was suspended in 200 µL of 80% methanol, mixed vigorously, and sonicated for 20 min at room temperature. Supernatant was filtered through a 0.45 µm filter after centrifugation at 13,000 rpm for 5 min. Subsequent analysis was conducted employing a flame ionization detector (HP-5890/5971, Hewlett Packard, Palo Alto, CA, USA) in conjunction with a GC column (DB-FFAP 123-3253, Agilent Technologies, Inc., Santa Clara, CA, USA, dimensions: 50 mm × 0.32 mm × 0.50 μm) Quantification of SCFAs levels was performed using acetic acid, propionic acid, and butyric acid as reference standards. The cecum SCFA content was determined via calibration curves constructed from these respective standards. The aforementioned organic acids (acetic, propionic, and butyric) were procured from Sigma-Aldrich (St. Louis, MO, USA).

### 2.9. Statistical Analysis

For the evaluation of experimental data, statistical analyses were conducted, and the findings are expressed as mean values accompanied by their standard deviations, derived from a triplicate set of experiments. To assess differences between experimental groups (*p* < 0.05 or *p* < 0.01 was considered statistically significant), the Kruskal–Wallis test was applied using GraphPad Prism 8 (GraphPad Software, San Diego, CA, USA).

## 3. Results

### 3.1. Evaluation of Intestinal Immune Responses to Oral Administration of KRG-P

**Figure 2:**
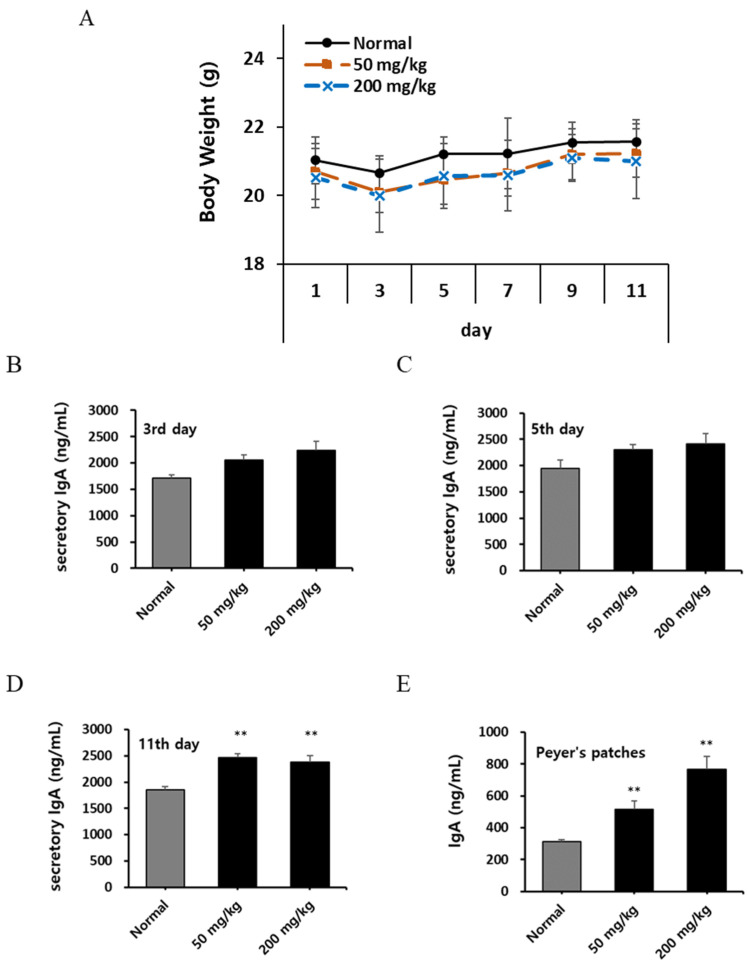
Effects of oral KRG-P administration on mouse body weight and IgA production in mouse feces. Five C3H/HeN mice per group were administered daily doses of KRG-P for 10 days. (**A**) Body weight was monitored every two days. (**B**–**D**) Feces collected on the 3rd, 5th, and 11th days were analyzed for IgA using a mouse ELISA kit. (**E**) Peyer’s patches were harvested from euthanized mice, and the cells were cultured for 5 days in a CO_2_ incubator. IgA levels were then assessed using a mouse ELISA kit. ** *p* < 0.01 versus the normal group.

The intestine, especially the small intestine with its extensive lumen, is exposed to external environmental factors and harbors numerous potentially pathogenic microbes. Peyer’s patches are dome-shaped cell aggregates located within the intestinal lumen that are critical for immune surveillance in the gut [23,24,25]. These patches predominantly facilitate immune responses within the mucosa of the jejunum and ileum and are the initial sites for immune reactions in the body. They are central to the production of IgA, which constitutes approximately 6% of all immunoglobulins in the body. IgA is a secretory antibody that plays a key role in eliminating pathogens that invade the mucosa by forming dimers with four antigen-binding sites [30,31]. In this study, C3H/HeN mice were used to assess the changes in IgA production following KRG-P administration for 10 days. Body weight was monitored every alternate day beginning from the 3rd day, and no significant weight loss was observed in any of the mice (Figure 2A). Fecal samples collected on days 3, 5, and 11 were dissolved in PBS and analyzed for IgA levels (Figure 2B). Although an increase in IgA levels was noted on the 3rd day in both the normal- and high-dose KRG-P groups, no significant changes were observed across all the groups (Figure 2B). A dose-dependent increase in IgA levels was observed on the 5th day, although this was not statistically significant (Figure 2C). However, a significant increase in IgA secretion was observed on the 11th day in the groups receiving 50 and 200 mg/kg KRG-P, compared to that in the control group (Figure 2D). Peyer’s patches were isolated from the small intestine 10 days post-administration of KRG-P, and immune cells were aseptically collected and cultured for 5 days. Analysis of the supernatants revealed a dose-dependent increase in IgA secretion in the Peyer’s patches (Figure 2E). Our results indicate that the oral administration of KRG-P improves IgA production in the intestine. IgA acts as the first line of defense in the intestinal mucosa, suggesting the potential of KRG-P to enhance mucosal immunity against pathogen invasion.

### 3.2. Analysis of Intestine mRNA Expression of α-Defensin and Lysozyme Following Oral Administration of KRG-P

**Figure 3:**
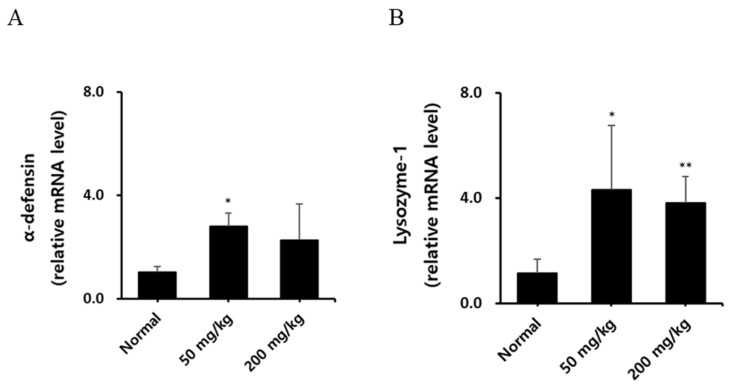
Effect of oral administration of KRG-P on intestinal mRNA expression of mice. Five C3H/HeN mice per group were orally administered with the indicated daily doses of KRG-P for 10 days. Eleven days after oral administration of KRG-P, the mice were euthanized. Intestinal tissues were collected from the jejunum region of the small intestine for RNA extraction. The relative mRNA expression of α-defensin (**A**) and Lysozyme (**B**). * *p* < 0.05, ** *p* < 0.01 vs. the normal group.

The intestine is exposed to various antigens, including food. It plays a crucial role in maintaining host intestinal homeostasis against foreign substances. To that effect, α-defensin, produced primarily by paneth cells in intestinal epithelial cells, possesses antimicrobial, antiviral, and antifungal activities [24,25]. This small peptide can disrupt microbial cell membranes. Similarly, lysozyme, found in tears, saliva, breast milk, and intestinal mucus, also has antimicrobial properties. After the oral administration of KRG-P, jejunum segments were harvested from mice to extract RNA. Real-time RT-PCR was used to analyze the mRNA expression of α-defensin and lysozyme. As shown in Figure 3, α-defensin mRNA expression increased in all KRG-P-treated groups compared to the control group. Notably, the 50 mg/kg group showed a statistically significant increase, while the 200 mg/kg group exhibited an even more pronounced increase. For lysozyme mRNA expression, significant increases were observed in both the 50 mg/kg and 200 mg/kg groups. The 50 mg/kg group showed statistical significance, while the high-dose group demonstrated higher significance. These findings suggest that KRG-P can modulate intestinal antimicrobial defense mechanisms in a dose-dependent manner. The marked increase in α-defensin and lysozyme mRNA expression at 50 mg/kg and 200 mg/kg indicates that KRG-P has the potential to enhance intestinal immune function.

### 3.3. Analysis of Intestinal Homeostasis and Strengthening of Intestinal Wall Proteins Following Oral Administration of KRG-P

**Figure 4:**
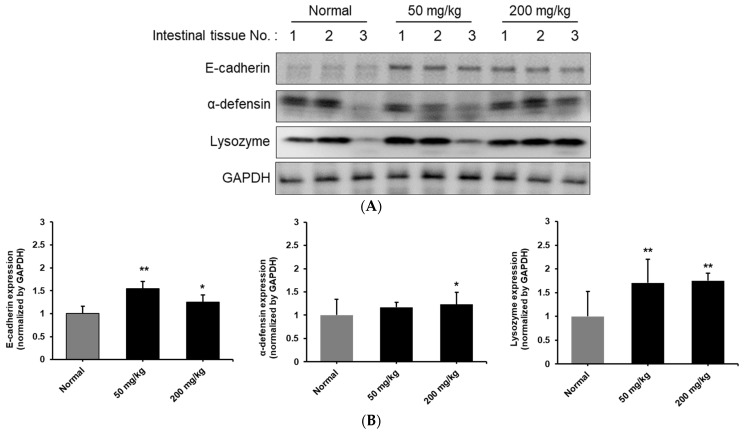
Effect of oral administration of KRG-P on E-cadherin, α-defensin, and lysozyme expression in small intestine tissue from mice. C3H/HeN mice were orally administered with the indicated daily doses of KRG-P for 10 days. (**A**) The protein level of E-cadherin, α-defensin and lysozyme were determined by immunoblotting with the specific antibody. Glyceraldehyde 3-phosphate dehydrogenase (GAPDH) was used as a loading control. (**B**) The bar chart quantifies the immunoblotting image. * *p* < 0.05, ** *p* < 0.01 vs. the normal group.

This study investigated the effects of oral KRG-P administration on the protein expression of E-cadherin, α-defensin, and lysozyme in small intestine tissue in Figure 4. Western blot analysis revealed that the E-cadherin expression was significantly increased in the 50 and 200 mg/kg groups of mice. The α-defensin expression was elevated in the KRG-P-treated groups, with considerable inter-individual variation observed. The 200 mg/kg group showed a more consistent increase compared to the 50 mg/kg group, suggesting a possible dose-dependent effect. Lysozyme expression showed a noticeable increase in both the 50 mg/kg and 200 mg/kg groups compared to the normal group, with the 200 mg/kg group showing a slightly more pronounced effect. GAPDH was used as a loading control, demonstrating consistent protein loading across all samples and validating the observed changes in protein expression. These results suggest that KRG-P administration may enhance the expression of tight junction proteins and antimicrobial peptides in the intestinal epithelium, although some individual variability was observed. In conclusion, these findings indicate that oral KRG-P administration may have positive effects on intestinal health. Increased E-cadherin suggests enhanced barrier function, while elevated α-defensin and lysozyme levels indicate improved antimicrobial defense. Overall, KRG-P shows potential for maintaining intestinal homeostasis and enhancing immune function.

### 3.4. Analysis of SCFAs in Cecum of Mice Following Oral Administration of KRG-P

Dietary fibers, including polysaccharides, are fermented by gut microbiota to produce short-chain fatty acids (SCFAs) such as acetate, propionate, and butyrate [32,33,34,35,36]. These SCFAs serve as primary energy sources for both the microbiota and the host, playing crucial roles in regulating the intestinal pH, cellular processes, and nutrition for intestinal epithelial cells [27]. SCFAs production has multiple beneficial effects as follows: it acidifies the gut environment, inhibits harmful bacteria growth, affects bile acid solubility, enhances mineral absorption, and reduces ammonia absorption [27,28]. These actions contribute to reducing risks of various gastrointestinal and systemic diseases while improving colon function [29,30]. As shown in Table 2, we analyzed the effects of the oral administration of KRG-P on the short-chain fatty acid (SCFA) profile in the cecum of mice. The results revealed changes in the SCFAs’ composition in the KRG-P-treated groups, with an increase in butyric acid concentration. Specifically, butyric acid levels were elevated to 6.3 ± 1.2 mM and 6.5 ± 1.0 mM in the 50 mg/kg and 200 mg/kg KRG-P groups, respectively, compared to the control. Acetic acid concentrations showed a transient increase (17.9 ± 2.1 mM) in the 50 mg/kg group but returned to control levels (15.2 ± 0.8 mM) in the 200 mg/kg group. Propionic acid concentrations did not exhibit significant changes across all experimental groups (range: 1.5–1.8 mM). These findings suggest that the oral administration of KRG-P may significantly influence the SCFA profile in the mouse cecum, particularly in butyric acid production. This implies there may be potential effects on the gut microbiota structure and overall intestinal homeostasis.

## 4. Discussion

Polysaccharides are macromolecules with complex structures composed of thousands of monosaccharides, which are difficult to digest by human digestive enzymes and are only partially metabolized by intestinal microbiota. The intestinal mucosa serves as a crucial barrier between the internal and external environments. Various components including tight junctions between intestinal epithelial cells, mucus layer, commensal bacteria, and antimicrobial proteins, such as lysozyme and defensins, provide non-specific defense. Additionally, the intestinal mucosal immune system offers specific immune responses that effectively eliminate previously encountered antigens.

In this study, we investigated the effects of Korean Red Ginseng Polysaccharides (KRG-P) on intestinal mucosal immunity, with the most notable finding being increased the IgA production through Peyer’s patches. After KRG-P administration, significant increases in IgA levels were observed both in immune cells from Peyer’s patches and in feces. IgA production in Peyer’s patches involves a series of processes starting with antigen sampling by M cells, followed by dendritic cell processing, T cell activation, and ultimately B cell differentiation into IgA-producing cells [23,24,25]. Cytokines such as APRIL and BAFF, secreted by intestinal epithelial cells, are known to play a crucial role in this process [25]. Secretory IgA (SIgA), the predominant antibody in intestinal mucosa, functions through various mechanisms. It structurally prevents pathogen attachment to epithelial cells and blocks infection by directly binding to receptor recognition domains. Furthermore, it contributes to intestinal microbial homeostasis by promoting commensal bacterial biofilm formation and inducing immune tolerance. These findings align with other studies, such as reports showing increased intestinal IgA and Peyer’s patch lymphocytes in immunosuppressed mice after seven days of lentinan administration [37] and increased salivary IgA in humans after 4–6 weeks of Chlorella pyrenoidosa supplementation [38].

The second finding is the enhancement of the innate immune barrier. Our results demonstrated an increased expression of the major antimicrobial peptides, α-defensin and lysozyme, which strengthen the host’s defense mechanism against pathogens. Similar results were reported in the study by Trachsel et al. (2019), where resistant potato starch was shown to enhance barrier function through increased expression of β-defensin 1, MUC2, IL-6, and IgA [39]. Notably, we observed an increased expression of E-cadherin, an adherens junction protein in the intestinal epithelium, which plays a crucial role in reinforcing barrier integrity. These changes collectively contribute to preventing harmful substances from penetrating the intestinal epithelium. The increased expression of these barrier proteins along with the enhanced E-cadherin expression indicates that KRG-P not only strengthens immune responses but also contributes to reinforcing both physical and chemical barriers of the intestinal epithelium. The increase in E-cadherin expression by KRG-P is thought to be regulated through various signaling pathways. In the Wnt/β-catenin pathway, KRG-P inhibits Wnt signaling, reducing β-catenin nuclear translocation, which can lead to the transcriptional activation of E-cadherin. Additionally, the TGF-β/Smad signaling pathway and phosphorylation of GSK-3β through the PI3K/Akt pathway are expected to contribute to the increased E-cadherin expression.

The third finding is the alteration of SCFA levels in the cecum, as shown in Table 2. The increased butyric acid levels demonstrate that KRG-P affects gut microbial composition and metabolism through several mechanisms. First, the polysaccharide components of KRG-P act as prebiotic substrates that promote selective fermentation by butyrate-producing bacteria such as Faecalibacterium prausnitzii and Roseburia species. Second, KRG-P creates a favorable intestinal environment for SCFA production by modulating intestinal pH and oxygen levels, which promotes the growth of beneficial bacteria, while inhibiting pathogenic bacterial proliferation. This dual action of KRG-P improves the balance of gut microbiota, resulting in increased SCFA production. Among the SCFAs, butyrate predominantly remains in the colon, providing energy to intestinal epithelial cells (IECs) or acting as a signaling molecule, while acetate and propionate are found in lower concentrations. Propionate mainly functions in the liver, aiding in gluconeogenesis via the gut-liver axis and reducing cholesterol production, while acetate circulates in the bloodstream and is metabolized by organs like the muscles, heart, and brain [40,41]. The increase in butyric acid is particularly noteworthy, as it serves as the primary energy source for colonocytes and promotes intestinal epithelial barrier function while suppressing colonic inflammation and colorectal cancer [42,43]. Furthermore, butyrate-producing bacteria like Faecalibacterium prausnitzii and Roseburia species are often reduced in inflammatory bowel disease (IBD) patients [44], suggesting that the KRG-P’s ability to enhance butyrate production may have therapeutic implications for intestinal inflammatory conditions.

Although this study was conducted in mice, these results provide important insights into exploring the potential benefits of KRG-P on human gut health. The observed effects, including the increased IgA production, enhanced antimicrobial peptide expression, improved barrier function, and SCFA regulation, suggest promising therapeutic potential. However, translating these preclinical findings to human applications requires careful consideration of interspecies differences such as gut microbiota composition, dietary patterns, and genetic factors.

## 5. Conclusions

This study demonstrated the effects of Korean red ginseng-derived polysaccharides (KRG-P) on gut health. Oral administration of KRG-P significantly increased IgA production in feces and Peyer’s patch cells, and enhanced mRNA and protein expression of α-defensin and lysozyme in small intestinal tissue. Moreover, the increased E-cadherin expression strengthened the intestinal barrier, and the improved short-chain fatty acid content was observed in the cecum. These results suggest that KRG-P modulates the gut immune system via Peyer’s patches. Increased IgA production indicates enhanced intestinal immunity, while elevated α-defensin and lysozyme expression signifies improved antimicrobial defense. E-cadherin upregulation suggests improved intestinal barrier integrity, and increased short-chain fatty acids imply improved gut microbiota balance and metabolic function. This suggests that KRG-P is not merely an immune stimulant, but a multifunctional agent capable of comprehensively regulating intestinal homeostasis. 

## Figures and Tables

**Table 1:** Specific primers sequences for real-time PCR.

	Forward (5′ -> 3′)	Reverse (5′ -> 3′)
α-defensin	TaqMan Mm02524428	
Lysozyme-1	CCAGGCCAAGGTCTACAATC	TGAGCTAAACACACCCAGTC
GAPDH	CTCATGACCACAGTCCATGC	CACATTGGGGGTAGGAACAC

**Table 2:** The contents of short chain fatty acids in cecum from KRG-P orally administered mice.

SCFAs	Normal	KRG-P 50 mg/kg	KRG-P 200 mg/kg
Acetic acid	14.0 ± 2.4	17.9 ± 2.1 **	15.2 ± 0.8
Propionic acid	1.7 ± 0.3	1.5 ± 0.1	1.8 ± 0.2
Butyric acid	4.1 ± 0.9	6.3 ± 1.2 **	6.5 ± 1.0 **

The cecum contents were collected from C3H/HeN mice following a 10-day oral administration of KRG-P at doses of 50 and 200 mg/kg per day. The normal group received the vehicle only. The cecum was immediately extracted using 80% methanol to preserve the metabolite profile. Short-chain fatty acids, including acetate, propionate, and butyrate, were quantitatively analyzed using gas chromatography coupled with flame ionization detection (GC-FID), as described in Section 2.8. ** *p* < 0.01 vs. the normal group.

## Data Availability

The original contributions presented in the study are included in the article, further inquiries can be directed to the corresponding author.

## Abbreviations

Korean red ginseng polysaccharides (KRG-P), Immunoglobulin A (IgA), Short chain fatty acids (SCFAs).
